# Prevalence and risk factors for asthma among children aged 0–14 years in Hangzhou: a cross-sectional survey

**DOI:** 10.1186/s12931-016-0439-z

**Published:** 2016-09-27

**Authors:** Dan Xu, Yingshuo Wang, Zhimin Chen, Shuxian Li, Yungai Cheng, Li Zhang, Lingzhi Zhao

**Affiliations:** 1 Department of Pulmonology, The Children’s Hospital of Zhejiang University School of Medicine, 3333 Binsheng Road, Hangzhou, 310003 Zhejiang Province China; 2 Department of Internal Medicine, Hangzhou Children’s Hospital, 201 Wenhui Road, Hangzhou, 310014 Zhejiang Province China; 3 Department of Pediatric, Yueqing People’s Hospital, 338 Qingyuan Road, Yueqing, Wenzhou, 325600 Zhejiang Province China

**Keywords:** Asthma, Children, Epidemiology, Prevalence, Trigger, Risk factor, Inhaled corticosteroid, Exclusive breastfeeding

## Abstract

**Background:**

Asthma is a global problem. Prevalence varies among different countries and cities. We aimed to obtain the prevalence, describe the characteristics, and discover factors that may relate to asthma in Hangzhou.

**Methods:**

This cross-sectional study was conducted in Hangzhou. The subjects were children aged 14 years and younger. A control group of non-asthma children that matched in age and sex with each asthmatic patient was also randomly selected and interviewed. International Study of Asthma and Allergies in Childhood and National Epidemiology study of Asthma and Allergies in China questionnaires were used in this survey.

**Results:**

We have questionnaired 13,877 children, and 665 (4.8 %) children were diagnosed asthma. The guardians regarded the cost of asthma management affordable in 49.4 %, tolerable in 46.9 %, and intolerable in 3.7 %. Both guardians and children have been absent from work or school due to children’s asthma.

Respiratory tract infection was the most common trigger of asthma attacks (85.1 %). Other common causes included cold air, house dust, exercise, fish and shrimp, pollen, and et al. Interestingly, we also found in children 6 years and older, some triggers happened more than that in children 5 years and younger. Those factors included exercise, emotional changes, house dust, pollen, renovation works in the home, mosquito–repellent incense and pets (all the *p* values were <0.05).

We compared some factors may relate to asthma development. Higher percentage of family history of asthma, personal history of allergy (atopic dermatitis, drug allergy and food allergy), comorbidities (allergic rhinitis, sinusitis, adenoidal hypertrophy, and urticaria), caesarean birth and complications ever happened during pregnancy were discovered in asthma children than in non-asthma children (all the *p* values were <0.05). Exclusive breastfeeding within first 6 months and keeping animals had higher percentage in non-asthma children than in asthma (both the *p* values were <0.05).

Inhaled corticosteroid (ICS)/ICS + long-acting beta2 agonists (LABA) was applied to 46.2 % of patients. Traditional Chinese medicine (TCM) was used in 44.2 % of asthma children, while leukotriene receptor antagonist (LTRA) was used in 36.4 % of them. The adherence scored higher in TCM than in ICS/ICS + LABA (*P* = 0.003) and LTRA.

**Conclusions:**

In conclusion, we conducted an epidemiology study in Hangzhou. The prevalence of childhood asthma was 4.8 %. Asthma was an economic and social burden to both children and guardians. Risk factors of asthma development may include caesarean birth, personal history of allergy and concomitant allergic diseases. Exclusive breastfeeding within first 6 months and keeping animals might be protecting factors. TCM was really popular in China besides ICS/ICS + LABA and LTRA.

## Background

Asthma is characterized by variable symptoms of wheeze, shortness of breath, chest tightness and/or cough, and by variable expiratory airflow limitation, causing poor quality of life, emotional disorders, missing of schooling days and parents’ working days, and economic burdens. It is a common, chronic respiratory disease affecting 1–18 % of the population in different countries [1]. Prevalence varies among different countries and cities. The prevalence of childhood asthma in China increased from 0.93 % in 1990 to 1.54 % in 2000 [2]. However, exact date on prevalence of asthma in Hangzhou has not been reported yet. In this study, we aimed to obtain the prevalence of childhood asthma in Hangzhou, describe the characteristics of childhood asthma, and discover factors that may relate to asthma in Hangzhou.

## Methods

**Fig. 1:**
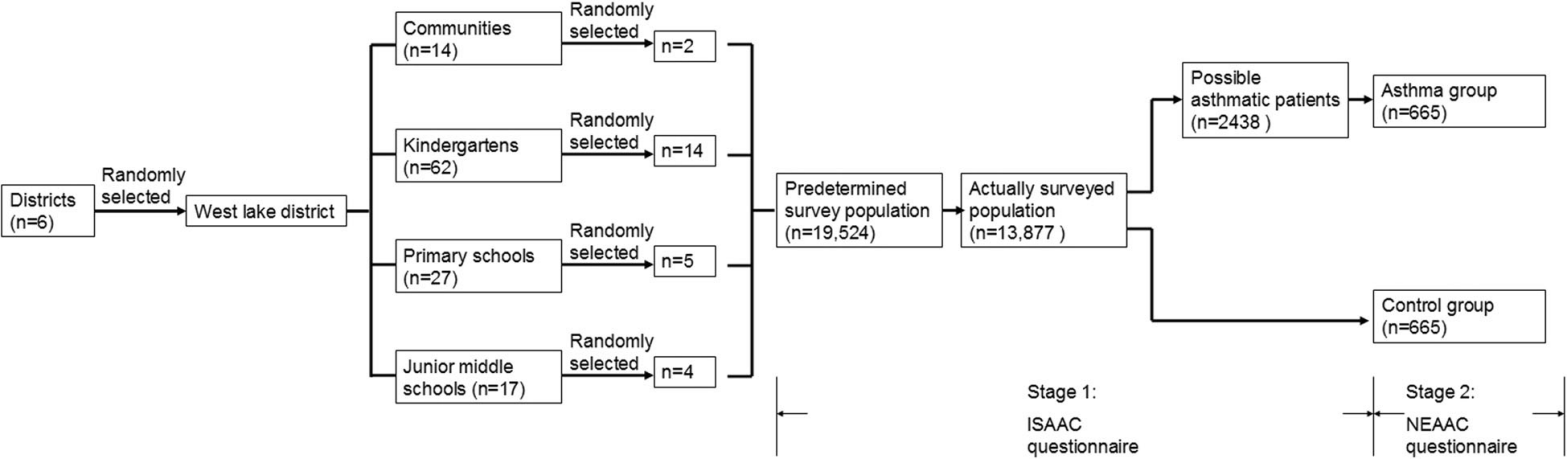
The sampling and investigation procedure: In the sampling stage, we firstly randomly selected one district, then randomly selected two communities, 14 kindergartens, five primary schools, and four junior middle schools. International Study of Asthma and Allergies in Childhood (ISAAC) and National Epidemiology study of Asthma and Allergies in China (NEAAC) questionnaires were used in stage 1 and stage 2 investigations, respectively

This cross-sectional study was conducted in Hangzhou, the capital of Zhejiang Province in the southeast coast of China. The predetermined sample size was 12,000 subjects, calculating from prevalence of 1.54 % in 2000 [2]. The subjects were children born from 1st, July, 1996 to 30th, June, 2010, and living in Hangzhou at least half a year for children older than 6 months, or lived in Hangzhou since born for children younger than 6 months. We randomly selected two communities, fourteen kindergartens, five primary schools, and four junior middle schools using stratified cluster sampling method (Fig. 1).

International Study of Asthma and Allergies in Childhood (ISAAC) and National Epidemiology study of Asthma and Allergies in China (NEAAC) questionnaires were used in this survey. In the first stage, the ISAAC questionnaires were filled in by all the children or their parents from selected communities, kindergartens, primary schools, and grade one in junior middle schools. If any of the answers to questions of “wheeze, ever”, “wheeze, last 12 months”, “‘he, he’ sound, last 12 months”, or “diagnosis of asthma, ever” were positive, the children were included as possible asthma patients. They were identified immediately by doctors on spot or called back by phone to doctors in the hospitals in charge of this investigation to make sure the diagnosis of asthma according to Global Initiative for Asthma (GINA) 2010 [3].

In the second stage, asthma patients were interviewed again. In-depth questionnaires (NEAAC questionnaire) were filled in by investigators. In addition, a control group of non-asthma children that matched in age and sex with each asthmatic patient individually was also randomly selected and interviewed. If the selected control child was not interviewed because of guardian’s refusal or other reasons, a second control would be selected and interviewed. NEAAC questionnaire were completed by investigators. The Survey started in September 2010 and finished in April 2011.

The study was approved by the Ethics Committee of Children’s Hospital, Zhejiang University School of Medicine. Each questionnaire was approved by the child’s guardian. Investigators were doctors or medical students, who were trained before the program. The questionnaires were reviewed in completeness and logicality, and were demanded to be revised properly by interviewing again.

Data were double entered using epi-info system. Data skewed distributed were expressed as medians (25th–75th interquartile ranges). Data normal distributed were expressed as mean ± standard deviation. McNemar test was applied for data comparison between asthma and control groups. Mann-Whitney U test was applied for quantitative data comparison between subgroups in asthma children. Chi-square test was applied for qualitative data comparison between subgroups in asthma children. Variables were considered statistically significant at *P* values of less than 0.05 by using two-sided tests. Data were analyzed by SPSS 19.0 (SPSS Inc., USA).

## Results

### Prevalence of childhood asthma

**Fig. 2:**
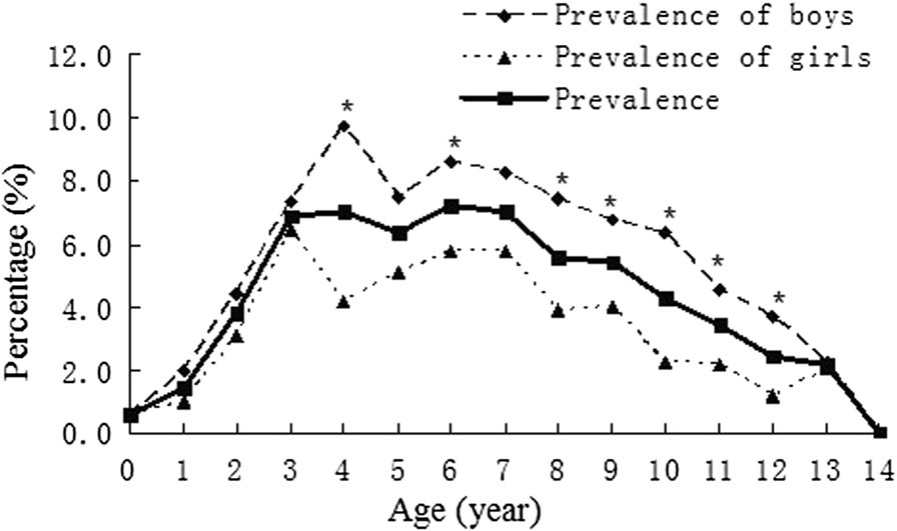
The prevalence of asthma children: The prevalence of asthma children was high in age 3 to 7. Prevalence of boys was higher than that of girls in age 4, 6, 8, 9, 10, 11 and 12, marked with an asterisk (all the *P* values were <0.05)

We have actually questionnaired 13,877 children. A total of 2438 possible asthma patients were selected and 665 (4.8 %) children were diagnosed asthma. Among asthma children, 34 (5.1 %) children were diagnosed cough variant asthma. The male to female ratio was 1:0.58 (420:245). The mean age was 6.9 ± 2.9 year. The prevalence of asthma children was higher in boys than that in girls in some ages (Fig. 2). Asthma children were divided into two groups, 0–5 years (*n* = 210), 6–14 years (*n* = 455).

A total of 665 children matched 1:1 in gender and age with each asthma child composed the control group.

### Economic and social impact

**Fig. 3:**
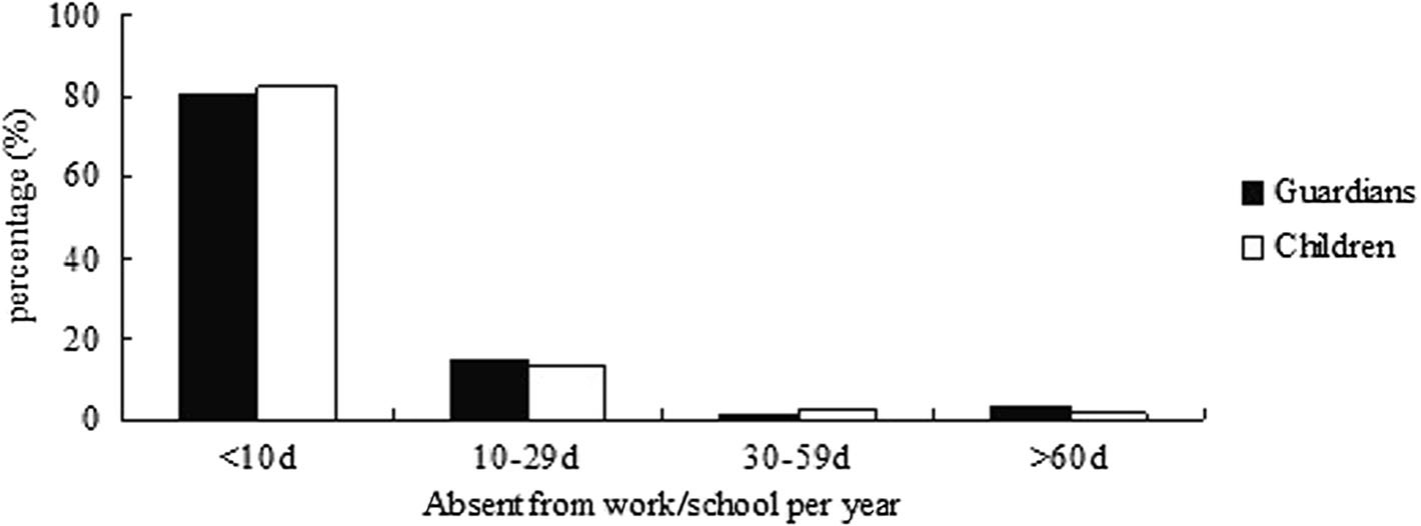
The percentage of different days absent from work/school: Both children and guardians are absent from work or school due to children’s asthma

We investigated asthma-related expenditure, which included all the expenditures spent on medication and devices, scheduled and unscheduled medical consults, hospitalization, blood and diagnostic tests, education, and lost productivity for the caretaker of asthmatic children, et al. Families spent less than 2000 yuan (41.9 %), 2000–5000 yuan (32.9 %), 5000–10,000 yuan (18.9 %), 10,000–30,000 yuan (5.4 %) and more than 30,000 yuan (0.9 %) in the year of spending most on asthma-related expenditures. The family yearly incomes were less than 60,000 yuan (14.2 %), 60,000–120,000 yuan (44.5 %), 120,000–240,000 yuan (25.15 %) and more than 240,000 yuan (16.2 %). The guardians regarded the cost affordable in 49.4 %, tolerable in 46.9 %, and intolerable in 3.7 %. Both guardians and children have been absent from work or school due to children’s asthma (Fig. 3). Fortunately, none of the children was suspended from school for asthma. About 82.2 % of these children attended physical education classes normally. However, 17.3 % of them took part in PE classes selectively while 0.5 % never attended PE classes.

### Clinical characteristics and triggers

In this study, the most common symptom was wheezes (91.1 %), followed by cough (85.4 %), shortness of breath (34.3 %), nocturnal waking (16.8 %), and chest tightness (16.1 %).

In 85.1 % asthmatic children, respiratory tract infection was a trigger of asthma attacks. Other causes included cold air (53.1 %), house dust (20.3 %), exercise (14.6 %), fish and shrimp (14.0 %), pollen (11.1 %), et al. (Table 1). Interestingly, we also found in children 6 years and older, some triggers happened more than that in children 5 years and younger. Those factors included exercise (16.9 % vs. 9.5 %, *p* = 0.012), emotional changes (4.8 % vs. 1.4 %, *p* = 0.032), house dust (23.1 % vs. 14.3 %, *p* = 0.009), pollen (13.6 % vs. 5.7 %, *p* = 0.003), renovation works in the home (11.0 % vs. 3.8 %, *p* = 0.002), mosquito–repellent incense (6.4 % vs. 2.4 %, *p* = 0.030) , and pets (4.4 % vs. 1.0 %, *p* = 0.021). Five kinds of triggers were inhaled causes.

**Table 1:** The triggers of childhood asthma in Hangzhou

Triggers	Percentage (%)
Daily activities	
Respiratory tract infection	85.1
Cold air	53.1
Exercise	14.6^a^
Tied	7.1
Emotional changes	3.8^a^
Inhaled causes	
House dust	20.3^a^
Pollen	11.1^a^
Renovation works in the home	8.7^a^
Smoke	7.8
Paint	6.8
Moldy smell	6.8
Odor of cooking oil	6.2
Mosquito–repellent incense	5.1^a^
Pets	3.3^a^
Perfume	3.0
Disinfectant	0.9
Food and medicine	
Fish and shrimp	14.0
Egg	4.2
Milk	3.5
Peanut	2.0
Other nuts	1.5
Fruit	1.2
Beans	1.1
Wheat	0.6
Vegetables	0.6
Aspirin	0.3
Others	3.8

^a^In children 6 years and older, those triggers happened more than that in children 5 years and younger (*P* < 0.05)

### Risk factors for asthma development

We compared some factors may relate to asthma development (Table 2).Higher percentage of family history of asthma, personal history of allergy (atopic dermatitis, drug allergy and food allergy), and comorbidities (allergic rhinitis, sinusitis, adenoidal hypertrophy, and urticaria) were discovered in asthma children than in non-asthma children (all the *P* values were <0.05).

**Table 2:** Factors may relate to asthma

	Asthma^a^	Non-asthma^a^	*P* value
Family history			
Asthma in parents	10.1 (67/665)	1.7 (11/665)	<0.001
Birth history			
Preterm birth	9.5 (31/326)	7.1 (46/644)	0.101
Caesarean birth	62.6 (201/321)	45.7 (297/650)	<0.001
Complications during pregnancy	23.6 (109/461)	14.0 (64/458)	<0.001
Infections during pregnancy	2.8 (13/462)	0.9 (4/458)	0.057
Past history			
Exclusive breastfeeding	51.2 (166/324)	63.2 (405/641)	0.002
Hospitalization during neonatal	14.0 (65/463)	12.9 (59/458)	0.634
Drug allergy	51.9 (136/262)	10.3 (68/658)	<0.001
Food allergy	13.6 (53/389)	6.5 (43/663)	0.001
Atopic dermatitis	37.1 (93/251)	4.3 (28/658)	<0.001
Comorbidities			
Allergic rhinitis	79.0 (319/404)	11.2 (74/658)	<0.001
Sinusitis	26.2 (56/214)	4.6 (30/657)	<0.001
Adenoidal hypertrophy	40.6 (99/244)	2.9 (19/657)	<0.001
Urticaria	47.5 (135/284)	9.6 (63/658)	<0.001
Indoor environment			
Renovation works in the home after child’s birth	47.7 (155/325)	35.8 (224/626)	0.060
Carpet using	8.4 (27/322)	6.8 (43/632)	0.551
House plants	44.7 (144/322)	46.0 (294/639)	0.335
House animals	9.2 (30/325)	15.0 (97/647)	0.013

^a^Data were expressed as percentage (%) (number of positive children/number of children had data )

Additionally, higher percentages of caesarean birth and complications ever happened during pregnancy were found in asthma than in non-asthma (both the *P* values were <0.05).

Exclusive breastfeeding within first 6 months and keeping animals, including cats, dogs, birds, pigs, horses, cows, et al. had higher percentage in non-asthma children than in asthma (both the *P* values were <0.05).

### Current situation of asthma therapy

**Fig. 4:**
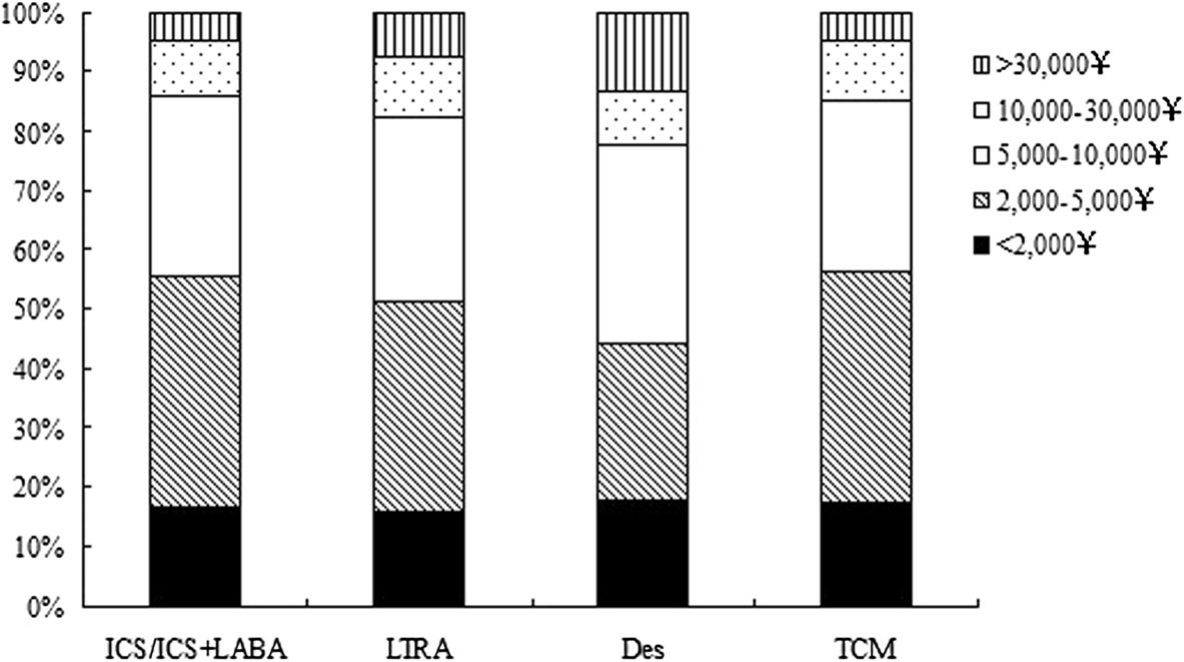
The most expensive yearly cost of asthma in different therapies ever used as controller treatment. Data were expressed as proportions. No significant statistical differences were found between each two controller groups (all the *P* values were >0.05). ICS/ICS + LABA = inhaled corticosteroid/inhaled corticosteroid + long-acting beta2 agonists, LTRA = leukotriene receptor antagonist, DES = desensitization therapy, TCM = traditional Chinese medicine

The therapies ever used as controller treatment were inquired. On the top of the list, inhaled corticosteroid (ICS) or ICS + long-acting beta2 agonists (LABA) was applied to 46.2 % of patients. As Chinese people, traditional Chinese medicine (TCM) using in asthma patients was the second most frequently used (44.2 %). And the third one was leukotriene receptor antagonist (LTRA) (36.4 %). Some have tried desensitization therapy (7.2 %). The ages of patients who ever used LTRA, ICS/ICS + LABA, TCM and desensitization therapy were 6.7 ± 2.9, 7.0 ± 2.8, 7.4 ± 3.0, and 8.3 ± 2.6, separately. Significant statistical differences were found in ages between every two controller groups (all the *P* values were <0.05). Although patients ever used desensitization therapy seemed to have more proportion of more than 30,000 yuan (12.5 %) of the most expensive yearly cost of asthma (Fig. 4), we did not found any significant statistical differences among these controller groups (all the *P* values were >0.05).

Adherence was assessed by parents using a scale from 0 to 10 points, which indicating not following doctor’s advice at all to completely following doctor’s advice. For ICS/ICS + LABA, TCM, LTRA, and desensitization therapy, the adherence scored 10 (8–10), 10 (10–10), 10 (8–10) and 10 (9–10), respectively. Data were expressed as medians (25th–75th interquartile ranges). We also found the adherence scored higher in TCM than in ICS/ICS + LABA (*P* = 0.003) and LTRA (*P* = 0.006). No statistical significance was found between other groups.

We explored some factors which we think may affect adherences of different therapies. Those factors included maternal or paternal education and income. We found only maternal education was related to adherence of ICS. Mothers holding college or above degrees had poorer adherence of ICS than those holding middle school or below degrees (*P* = 0.021). Maternal education did not affect adherences of other therapies. Paternal education and monthly income was not related to adherence of asthma medicine (all the *P* values were >0.05).

We also questionnaired reasons for not following doctor’s advice. Worrying about potential adverse effects (25.1 %) was the reason on the top of the list. However, only 2.7 % of patients actually showed adverse effects, such as uncomfortable in throat or mycotic stomatitis. No major adverse effects or mortality occurred in our study. The second reason was parents believing it was the time to cease medicine for well-controlled asthma (24.8 %) (Table 3).

**Table 3:** Reasons for not following doctor’s advice

Reasons	*n*	Percentage (%)
Worrying about potential adverse effects	131	25.1
Believing it was the time to cease medicine for well-controlled asthma	129	24.8
Forgotten	42	8.1
Believing the medicine not working	31	6.0
Adverse effects occurred	14	2.7
Others	49	9.4

## Discussion

In this study, we found the prevalence of childhood asthma was 4.8 % in Hangzhou. This data was higher than the nationwide prevalence of 1.54 % about 10 years ago [2]. The prevalence of asthma was highest in children aged 3 to 7 in this study (Fig. 2). High prevalence is related to high incidence. Prevalence and incidence of asthma was reported differently in different studies. About 80 % of asthma has onset by age 5 years [4]. Incidence of asthma was high in children aged 1 to 9 in a study [5]. In general, incidence rates are higher in children below 5 years [4, 6]. It is worth noting that the diagnostic criteria in the above literatures were not the same. Prevalence or incidence of a disease is based on its diagnosis. It may be difficult to make a confident diagnosis of asthma in children 5 years and younger, because episodic respiratory symptoms such as wheezing and cough are also common in children without asthma [1]. Because of this uniqueness, GINA always made ‘diagnosis and management of asthma in children 5 years and younger’ separately. However, in the practice of asthma management, misdiagnosis of asthma does occur. Close follow-up should always be recommended to reexamine the treatment or even the diagnosis itself. Incidence rates for boys were higher than for girls before 9-year-old while incidence rates for boys were lower than for girls after 9-year-old [7]. We found the prevalence of asthma in boys was higher than in girls before adolescence, which is in agreement with results from other studies [6].

It should be noticed that, as a developing country, about a half of the family yearly income were 60,000–120,000 yuan in our study. However, in the most costive year, asthma-related expenditure would be as high as more than 30,000 yuan in 0.9 % of families. That was why 3.7 % of guardians regarded the cost intolerable. In a systematic review, authors showed the mean annual cost/person, including direct and indirect cost on asthma management, was 151 to 4158 US dollars [8]. The cost of asthma was found to be strongly correlated with comorbidities, age, severity of disease, and some other factors [8]. We believe better control of asthma will help to decrease the cost of asthma.

Increased prevalence and heavy burden make early diagnosis and early treatment of asthma really important. In this study, we explored some factors that may be important for asthma development. We found the percentages of caesarean birth in asthma were higher than that in control group. In our country, the caesarean section rate rose dramatically from 3.4 % in 1988 to 39.3 % in 2008 [9]. Results about caesarean birth and asthma are conflicting. Caesarean birth was found associated with atopic diseases by altering exposure to maternal flora [10, 11]. In a meta-analysis, authors found a 20 % increase in the subsequent risk of asthma in children who had been delivered by Caesarean section [11]. However, childhood asthma in Malaysian children was not associated with delivery by caesarean section [12]. A recent study showed the previously reported association between caesarean birth and atopic disease may be due to confounding [13]. Whether the increasing rates of caesarean birth in China could partly explain the concomitant rise in asthma prevalence should be further demonstrated.

For children already diagnosed asthma, how to prevent the onset of acute asthma is still an interesting topic. In this study, we found respiratory tract infection was still a very important trigger of asthma attacks. Cold air, house dust, exercise, sea food and pollen were also important triggers. A significant negative correlation between asthma hospitalizations and daily mean temperature was found in an 8-year study [14]. Here we also found in children 6 years and older, some triggers happened more than that in children 5 years and younger. Most of them were inhaled triggers. This age-related difference should also raise the attention of patients and their parents to avoid the triggers.

We found concomitant allergic diseases, including atopic dermatitis, food allergy, allergic rhinitis, and urticaria had higher percentages in asthma children than in non-asthma children. Similar results were found in other literatures [15–19]. Asthma and rhinitis frequently coexist in the same subjects [20]. The prevalence of allergic rhinitis has generally increased in both adults and children over the last two decades in China [21]. Other allergic diseases had no exact data in comparing the prevalence in past decades in China. Further more, we found asthma children had higher probability that he or she once had one of these conditions, including drug allergy, food allergy and atopic dermatitis. So treat comorbidities like allergic rhinitis may decrease the possibility to develop to asthma or acute onset.

We also found exclusive breastfeeding within first 6 months and keeping animals had higher percentage in control group than in asthma patients. Most of results about breastfeeding and asthma encouraged breastfeeding. There were evidences that breastfeeding is protective for asthma (5–18 years) [22]. Breastfeeding, especially exclusively breastfeeding, was protective of asthma in Aboriginal children, which is consistent with what has been observed in non-Aboriginal children in Canada [23]. Three or more months of exclusive breastfeeding reduced the risk of asthmatic symptoms in the offspring of Latinas [24].

The treatment of ICS/ICS + LABA as a controller treatment was only used in less than a half in this study. The proportion of ICS/ICS + LABA using was lower than most studies in other areas [6]. This could be resulted from two reasons. One was that most of the patients were with mild asthma that only need treatment step 1, which means only as-needed short-acting beta-agonist. However, the severity of asthma was not included in this study. The other reason was inadequate ICS treatment as recommended by GINA [1]. Inadequate ICS is one of independent risk factors for asthma exacerbations and also a risk factor for developing fixed airflow limitation [1]. Many parents refused to use corticosteroid even in inhaled way, for fear the potential adverse effects (Table 3). Poor inhaler technique obviously happens more often in children than in adults. The use of ICS, especially in small children, need proper devices and trained skills. Grandparents take care of children in most families in China. In those families, the complicated process of ICS using always make the family want to cease the medicine. Theses reasons may contribute to inadequate use of ICS/ICS + LABA in asthma children. LTRA, the optional orally intake medicines [1] occupied 36.4 % of the treatment medicine. We found TCM was popular in treating asthma in our country. Use of TCM ranked the second place in asthma patients. In addition, we also found the adherence scored higher in TCM than in ICS/ICS + LABA and LTRA. Chinese herbal remedies for asthma such as anti-asthma herbal medicine intervention, modified Mai Men Dong Tang, Ding Chuan Tang et al. are used commonly in China. Since 2005, English language publications have reported results of double-blind, placebo-controlled clinical studies investigating efficacy and safety of TCM for asthma. Evidences from clinical studies have supported beneficial effects of TCM herbal therapy on asthma [25].

There were some limitations to our study. In investigating of side effects, we could not include the effect of height or body weight growth as this was a cross-sectional survey. Additionally, in answering some questions about things happened many years ago, recall bias occurred. This happened more easily in big children when answering about pregnancy, breastfeeding, and so on. In this study, we did not include the severity assessment which could have helped us to analyze some important aspects like the choose of therapies.

## Conclusions

In conclusion, we conducted an epidemiology study in Hangzhou. The prevalence of childhood asthma was 4.8 %. Asthma was an economic and social burden to both children and guardians. Respiratory tract infection was the most common trigger of asthma attacks (85.1 %). Other common causes included cold air, house dust, exercise, fish and shrimp, pollen, and et al. In children 6 years and older, some triggers happened more than that in children 5 years and younger. The triggers were exercise, emotional changes and five inhaled causes, including house dust, pollen, renovation works in the home, mosquito–repellent incense, and pets. Risk factors of asthma development may include caesarean birth, personal history of allergy (atopic dermatitis, drug allergy and food allergy), and concomitant allergic diseases (atopic dermatitis, food allergy, allergic rhinitis, and urticaria). Exclusive breastfeeding within first 6 months and keeping animals might be protecting factor of asthma development. The used of ICS/ICS + LABA, TCM, LTRA, and desensitization therapy was 46.2 %, 44.2 %, 36.4 % and 7.2 %. The adherence scored higher in TCM than in ICS/ICS + LABA and LTRA.

## Abbreviations

GINA: Global Initiative for Asthma
ICS: Inhaled corticosteroid
ISAAC: International Study of Asthma and Allergies in Childhood
LABA: Long-acting beta2 agonists
NEAAC: National Epidemiology study of Asthma and Allergies in China
TCM: Traditional Chinese medicine
LTRA: Leukotriene receptor antagonist
